# Supplementary material for: Inflammatory factors and risk of lung adenocarcinoma: a Mendelian randomization study mediated by blood metabolites
Source: Front Endocrinol (Lausanne). 2024 Aug 27;15:1446863. doi: 10.3389/fendo.2024.1446863 (PMC11384989; doi:10.3389/fendo.2024.1446863)
Supplement: Supplementary file 1 [file DataSheet1.zip › supplementary files/S2/GCST90200434/forest.pdf]

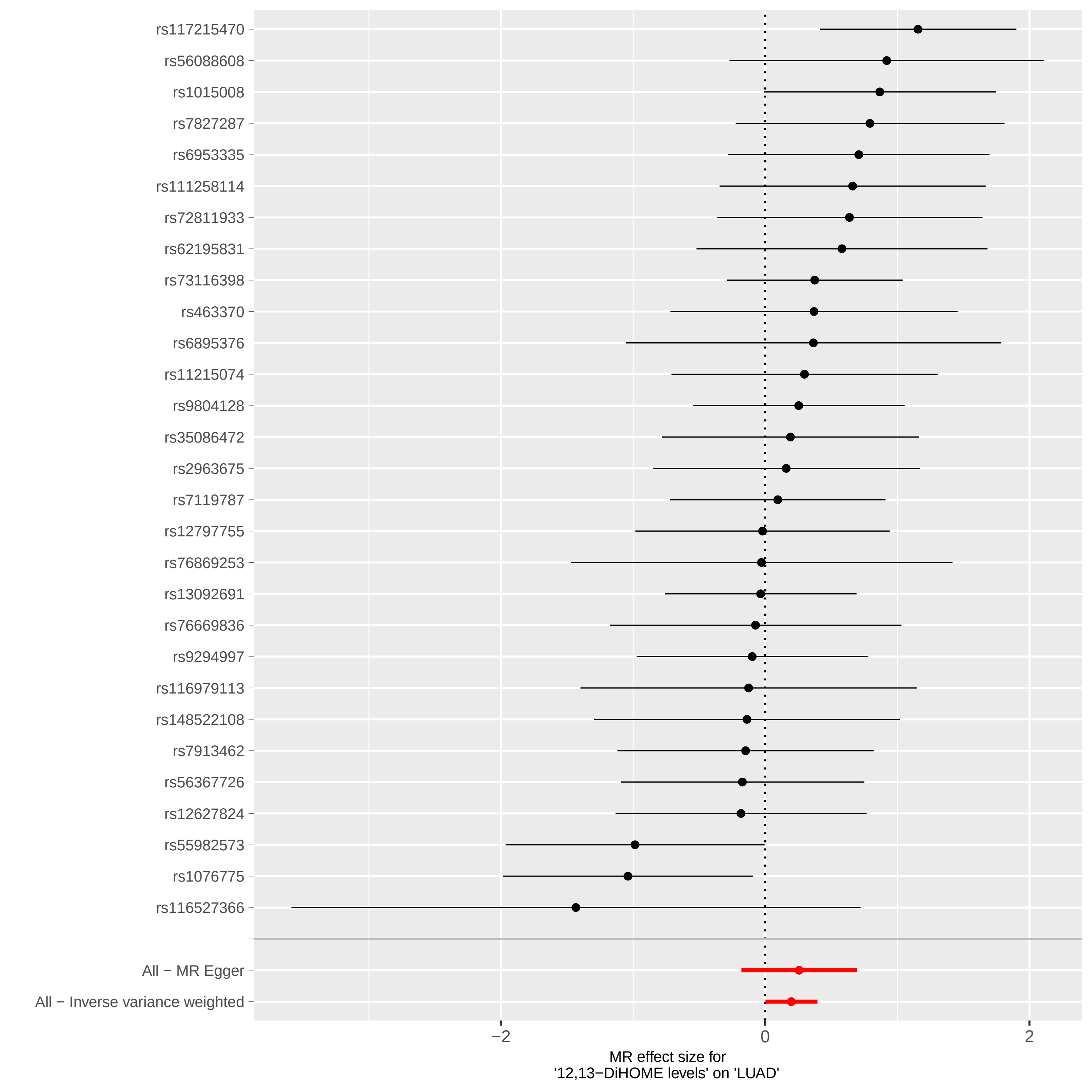
rs117215470
rs56088608
rs1015008
rs7827287
rs6953335
rs111258114
rs72811933
rs62195831
rs73116398
rs463370
rs6895376
rs11215074
rs9804128
rs35086472
rs2963675
rs7119787
rs12797755
rs76869253
rs13092691
rs76669836
rs9294997
rs116979113
rs148522108
rs7913462
rs56367726
rs12627824
rs55982573
rs1076775
rs116527366
All − MR Egger
All − Inverse variance weighted
−2
0
2
MR effect size for
'12,13−DiHOME levels' on 'LUAD'
